# Interaction of Polystyrene Nanoplastic with Lipid Membranes

**DOI:** 10.1021/acs.jpcb.5c00738

**Published:** 2025-04-10

**Authors:** Grzegorz Łazarski, Natan Rajtar, Marek Romek, Dorota Jamróz, Michał Rawski, Mariusz Kepczynski

**Affiliations:** † Jagiellonian University, Faculty of Chemistry, Gronostajowa 2, Kraków 30-387, Poland; ‡ Doctoral School of Exact and Natural Sciences, Jagiellonian University, Prof. S. Łojasiewicza 11, Krakow 30-348, Poland; § Department of Cell Biology and Imaging, Institute of Zoology and Biomedical Research, Jagiellonian University, 9 Gronostajowa Street, Kraków 30-387, Poland; ∥ National Synchrotron Radiation Centre SOLARIS, Jagiellonian University, 98 Czerwone Maki Street, Kraków 30-392, Poland

## Abstract

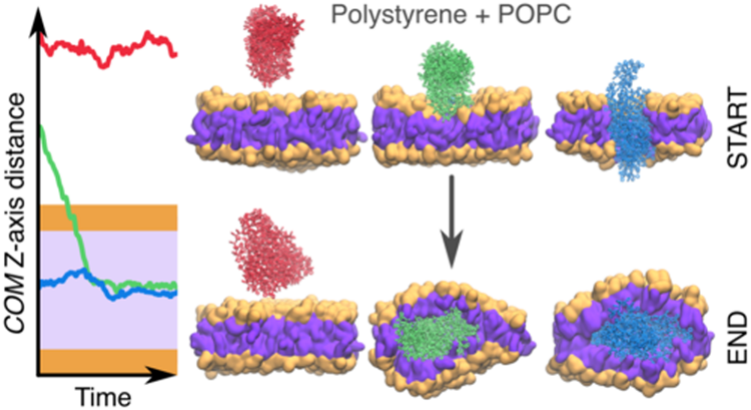

As demonstrated in *in vitro* studies, polystyrene nanoplastics (PSNPs) are effectively internalized by various cells. All known mechanisms of PSNP internalization involve the initial step of their interaction with the cell membrane, highlighting the importance of understanding such interactions at the molecular level. Here we consider the effects of PSNPs obtained from disposable food packaging on zwitterionic lipid membranes, used as a model system for protein-free cell membranes. We combined microscopic imaging and unbiased atomistic molecular dynamics (MD) to investigate the behavior of PSNPs on the surface and inside the lipid membrane. Our results show that PSNPs are hydrated and have a high negative surface charge when dispersed in an aqueous media. The penetration of PS nanoparticles into the lipid bilayer requires the removal of water molecules at the nanoparticle–membrane interface, which is an effective barrier to the entry of PSNPs into its hydrophobic region. Overcoming this energy barrier by slightly inserting the PS nanoparticle into the polar region of the membrane leads to its rapid penetration into the center of the bilayer and coating its surface with lipid molecules. PS nanoplastics do not disaggregate after penetrating the lipid membrane, which affects the molecular structure of the bilayer. In addition, our MD simulations demonstrated that small-molecule additives (e.g., unreacted monomers) present in nanoplastics can be released into lipid membranes if they are located close to the nanoparticle surface. The outcomes of this study are important for understanding the passive uptake of nanoplastics by cells.

## Introduction

Polystyrene (PS), particularly in its foamed form, is widely used to produce food and protective packaging, insulation materials, among other things.^1^ Due to the growing demand and widespread use, coupled with low recyclability, foamed PS accounts for the amount of waste released into the environment.^2^ For example, data from European countries indicate that the total waste generation of foamed PS from construction and packaging was about 530,000 tons in 2016/2017. The recycling rate of foamed PS waste in Europe in 2017 was 34% for single-use packaging waste and only 8% for construction waste.^3^ The remaining plastic debris can end up in the environment in various ways, where it is a major source of PS micro/nanoplastics (M/NPs) formed by photodegradation or mechanical damage. Despite numerous studies, the effects of PS M/NPs on living organisms or cells are not comprehensively understood. The toxicity of M/NPs to human cells has been studied.^4−7^ It is generally accepted that the smaller the particle, the higher the toxicity at lower concentrations. Although the exact mechanism of NP toxicity is unclear, it is assumed that they can penetrate the cell membrane and interfere with cell functions.^8^ Therefore, studying the interactions of PSNPs with model lipid membranes is of great importance to understand their effects on living organisms.

Interactions between bare PS and lipid membranes have been previously investigated using both molecular dynamics (MD) simulations and experimental methods. The permeation of dipalmitoylphosphatidylcholine (DPPC)/cholesterol (Chol) bilayers by PSNPs (composed of one polymer chain of different lengths) was studied as a function of nanoparticle size using coarse-grained (CG) MD simulations.^9^ Free energy calculations revealed that the penetration of PSNPs into the lipid bilayer is generally energetically favorable. NPs with diameters smaller than the membrane thickness are more easily absorbed into the hydrophobic interior of the lipid bilayers, where they do not cause noticeable disruption to the membrane integrity. In turn, Rossi et al. determined the effect of PSNPs on the properties of model lipid membranes using CG MD simulations.^10^ It was shown that PSNPs readily penetrate lipid membranes and disaggregate in their hydrophobic region. The dissolved PS chains alter the membrane structure, significantly reduce molecular diffusion, and soften the membrane. Yong used short 20 ns all atom (AA) simulations to study the initial stages of adsorption of a 100-unit PS oligomer to the 1-palmitoyl-2-oleoyl-*sn*-glycero-3-phosphocholine (POPC) lipid membrane.^11^ The simulations showed that although the polar lipid headgroups act as an effective barrier to prevent hydrophobic nanoparticles from interacting with the membrane surface, irreversible adhesion can be initiated by inserting the overhanging ends of the polymer chains into the hydrophobic interior of the membrane. Bochicchio et al. combined a broad spectrum of experimental techniques (calorimetry, X-ray, and neutron scattering) with MD united atom (UA) simulations to study the interaction between 25-unit PS oligomers and DPPC membranes (in liquid phase).^12^ The number of PS oligomers was shown to affect the thermotropic properties of the DPPC membrane. In addition, the presence of the polymer disrupts the structure and dynamics of the membrane in a concentration-dependent manner. CG MD simulations combined with fluorescence measurements were used to investigate the interactions between anionic PS nanoparticles and POPC liposomes.^13^ The results suggested that negatively charged PS NPs interact strongly with zwitterionic lipids, dissolving in the hydrophobic region of the membrane. Morandi et al. investigated the effect of PS oligomers (*M*_n_ = 500 Da, ∼5 units) on lipid bilayers composed of unsaturated lipids or a mixture of unsaturated/saturated lipids using a combination of scattering, fluorescence, and calorimetric techniques.^14^ It was shown that oligomers efficiently accumulate inside the membranes and affect their organization. In the gel phase, short PS chains are laterally segregated in the membrane. This segregation is caused by the poor solubility of the oligomers in the tightly packed region of the acyl chain. In the liquid phase, the oligomers are more evenly distributed and intercalate between the acyl chains. Incorporation of the oligomers changed the packing of lipids in the membrane in the liquid phase. On the contrary, Janke et al. recently showed using MD simulations that NPs from pristine PS with sizes smaller than 5 nm have a very low affinity for interaction with lipid membranes.^7^

In the present work, we use five different experimental techniques combined with atomistic molecular dynamics (MD) simulations to gain insight into the nature of the interaction of PS nanoparticles with lipid bilayers, which can improve our understanding of the interaction of PS nanoplastics with cellular membranes. Our research was particularly focused on determining penetration of PS nanoparticles into the membrane, its behavior inside the membrane, and its effect on the molecular organization of lipid molecules. To this end, we performed all atom MD simulations of several systems containing the PS nanoparticle and the zwitterionic lipid bilayer, used as a model of the cell membrane. As PSNPs may contain an unreacted monomer (styrene),^15^ a substance once considered a potential carcinogen,^16^ the release of monomer from the PS nanoparticles into lipid membranes was investigated using simulation methods. In the experimental part, we prepared PS nanoplastics from disposable food packaging and characterized their stability in aqueous environment. The penetration of the lipid membrane by PS NPs was observed directly using confocal microscopy. Brewster angle microscopy (BAM) and cryo-Electron Microscopy (cryo-EM) experiments were then used to recognize the miscibility of the PS material and lipids.

## Experimental Section

### Materials

Expanded polystyrene (EPS) foamed with the use of water vapor was acquired from take-out packaging certified for contact with food. 1-palmitoyl-2-oleoyl-*sn*-glycero-3-phosphocholine (POPC, ≥99.0%), sucrose (for molecular biology, ≥99.5%), glucose (for molecular biology, ≥99.5%), phosphate buffered saline (PBS, tablets), and Nile Red (NR, dye for microscopy) were purchased from Sigma-Aldrich and used as received. Tetrahydrofuran for HPLC (THF, ≥99.8%), chloroform for HPLC (≥99.8%) were purchased from Chempur. Dialysis tubes with 14,000 g mol^–1^ molecular cutoff were purchased from Sigma-Aldrich. All experiments were performed using the ultrapure Milli-Q water.

### Preparation of NR-Stained PSNPs

The EPS packaging (100 mg) was cut into smaller pieces and dissolved in THF (100 mL) in a glass bottle. For fluorescence staining, 1 mg of Nile Red (NR) was added at this stage. The solution was placed in a buret and poured at a constant speed into water stirred at 600 rpm. The dispersion was dialyzed against water for 2 days until THF was completely removed.

### Laser Scanning Confocal Microscopy (LSCM)

Giant liposomes (GUVs) were produced by electroformation, as previously described.^17^ Briefly, POPC was dissolved in chloroform to a concentration of 4 mg/mL. Twenty μL of the solution was evenly applied to two ITO glasses and the solvent was evaporated with a stream of inert gas. A chamber composed of two glasses and a 1 mm Teflon spacer was filled with a 0.2 M solution of saccharose. A 10 Hz AC field was applied with a voltage varying from 0.8 to 2 V in 0.4 V steps every 10 min and left for 1.5 h. The optical and fluorescence images were taken with a confocal Nikon A1-TiE microscope (Japan) equipped with a 488 nm laser and a NIS-Elements AR software, as previously described.^18^ 10 μL of the sample was placed between two coverslips.

### Dynamic Light Scattering (DLS) and Electrophoretic Light Scattering (ELS)

DLS and ELS measurements were carried out using a Zetasizer Ultra apparatus (Malvern Instrument Ltd., Malvern, U.K.), as previously described.^19,20^ The results were analyzed using the software provided by the manufacturer.

### Transmission Electron Microscopy (TEM)

Five-microliter droplets of PS nanoparticle suspension were applied onto 200 mesh Formvar-coated copper grids (Formvar/Carbon Supported Copper Grids, grid size 300 mesh, Sigma-Aldrich) and allowed to settle for 5 min. The grids were then blotted and imaged under a JEOL JEM2100 HT CRYO LaB6 (Japan) microscope operating at an accelerating voltage of 80 kV. Images were captured using a 4K × 4K CMOS camera (TVIPS—Tietz Video and Image Processing Systems, Gauting, DE) equipped with EMMENU4 software ver. 4.0.9.87.

### Cryo-Electron Microscopy (Cryo-EM)

The dry film rehydration method was used to prepare POPC liposomes containing 5 wt % of EPS. POPC (4 mg) was weighed into a vial, dissolved in a small amount of chloroform and 200 μL of EPS solution in chloroform (1 mg/mL) was added. A dry film was formed by blowing with a stream of nitrogen. The film was rehydrated with PBS buffer (2 mL) to obtain a lipid concentration of 2 mg/mL, using the procedure previously described.^21^ 3 μL of as-prepared dispersion was added on a Quantifoil 2/1 Cu mesh 200 microscope grid. Excess fluid was blotted, and the grid was plunge frozen in liquid ethane using a Vitrobot mk IV (Thermo Fisher Scientific). Freezing parameters were set as follows: blot force 3, blot time 3, humidity 100%, temperature 4 °C. The frozen grids were clipped and inserted into a Krios G3i cryo-Electron Microscope (Thermo Fisher Scientific) operating at an accelerating voltage of 300 kV and set to 105,000× magnification. The total dose deposited on the sample was around 40 e^–^/Å^2^. Imaging was performed using a K3 direct electron detector and a BioQuantum energy filter, both from Gatan.

### BAM Microscopy

Surface pressure (π) – molecular area (A) isotherms were recorded on a Langmuir trough KSV NIMA 5000 (KSV Instruments Ltd., Helsinki, Finland) at 25 ± 0.1 °C as previously described.^22^ Stock solutions of PS and POPC were prepared in chloroform, with deionized water used as the subphase. Brewster angle microscopy (BAM) images were recorded using an ultraBAM microscope (Accurion GmbH, Göttingen, Germany) equipped with a 658 nm laser and a 10× objective. The spatial resolution was 2 μm.

### Turbidimetric Measurements

Stability of the dispersions of polystyrene nanoplastics (PSNPs) was measured using a Turbiscan optical analyzer (TurbiSoft Classic 2.2.0.101, Formulaction, Toulouse, France). A cylindrical glass cuvette with a length of 10 cm and a diameter of 1.5 cm was filled with 5.7 mL of the dispersion (polymer concentration of 1 mg/mL). Radiation transmittance at λ = 850 nm along the dispersion in the cuvette was measured in three equal parts of the sample: bottom, middle, and top. The measurements were carried out for 168 h every 3 h.

## MD Simulations

### Overview

The composition of the simulated systems is listed in Table 1. We simulated three systems consisting of a POPC bilayer and one PS nanoparticle. A membrane containing 356 lipids in a hexagonal unit cell was prepared. The use of the hexagonal unit cell made it possible to reduce the simulated systems while providing opportunities to place the nanoparticle on the membrane at distances form the periodic boundaries reducing the likelihood of artifacts associated with the interaction of a nanoparticle with its own periodic image. As a model for polystyrene, we used 40-unit atactic polymers, corresponding to a molecular weight of about 4.2 kDa. Polymer nanoparticles (PSNPs) were prepared by placing 14 40-unit oligomers in a simulation box with water. To obtain a monomer-containing nanoparticle, 32 styrene (STYR) molecules were added at this stage. The energy of the systems was minimized, then the systems were equilibrated and simulated until an aggregate was formed. Systems **A** and **B** were prepared by placing PSNP at different distances relative to the bilayer midplane. In system **A**, the polymer aggregate was placed in the aqueous phase so that it did not directly contact the bilayer, while in system **B** it was partially immersed in the polar region of the membrane with almost no contact with the POPC hydrocarbon chains. The aggregate inserted into the bilayer in system **C** contained 32 STYR monomers encapsulated in the nanoparticle. The “membed” function^23^ available in GROMACS was used to prepare systems **B** and **C**. A few lipid molecules were removed during the PSNP insertion process (Table 1). System **A** was prepared by manually inserting the aggregate and removing water molecules. K^+^ and Cl^–^ ions were used to obtain an ionic strength of 0.14 M.

**Table 1:** Summary of the Simulated Systems^a^^b^

system	POPC molecules	PS aggregate	styrene molecules	distance from the center of membrane [nm]	simulation time [ns]
**A**	178 + 178	14 × 40 units		∼7	500
**B**	176 + 178	14 × 40 units		∼4	500
**C**	149 + 147	14 × 40 units	32	0	1000

a All simulations were performed at 298 K and an ionic strength of 0.14 M

b The table shows the number of lipid molecules (number of lipids in the upper and lower leaflets), styrene molecules, the distance of the center of mass of the PS aggregate from the bilayer center, and simulation time.

### Simulation Details

All simulations were performed using the GROMACS 2024 simulation package, with the exception of the membrane embedding step, which was performed using GROMACS 2019.^24^ The PS polymer and lipid molecules were parametrized in the CHARMM36 force field,^25^ and the TIP3P water model was used. Details of the parametrization of PS oligomers were described previously.^26^ The membrane was prepared using the CHARMM-GUI membrane builder, as described previously.^27^ The time step for all simulations was 2 fs. After the embedding step, each system was subjected to equilibration in the semi-isotropic isobaric–isothermal (*Np*_*xy*_*p*_*z*_*T*) ensemble for 500 ps. Pressure coupling was achieved using a Parrinello–Rahman barostat (τ = 5 ps).^28^ The reference pressure was 1 bar, and the compressibility was 4.5 × 10^–5^ bar^–1^. Production runs of varying length (Table 1) were carried out under the same *Np*_*xy*_*p*_*z*_*T* conditions. The temperature was kept constant at 298 K using the “V-rescale” thermostat, which is a modified Berendsen thermostat with a stochastic term added to reintroduce kinetic energy fluctuations.^23^ To avoid the “hot solute/cold solvent” problem, the temperature coupling was applied separately to the solvent, membrane, and other solutes. All hydrogen-containing bonds were constrained using LINCS.^29^ Dispersive interactions and short-range repulsion were described by a Lennard–Jones potential with a cutoff of 1.2 nm.

### Analysis of MD Simulations Results

All analyzes were carried out with in-house analysis scripts using the MDAnalysis library,^30^ with some routines based on code from the lipyphilic library.^31^ The need to write custom code was due to a lack of support for hexagonal unit cells, used in the simulated systems. Membrane thickness (*d*_P_), defined as the distance between the phosphorus atoms in the opposite leaflets,^32^ was calculated by retrieving the coordinates of the phosphorus atoms of each lipid along the membrane normal axis (the *Z* axis) from the trajectory. The data were then grouped based on which leaflet the lipid was part of (using the AssignCurvedLeaflets functionality provided by the lipyphilic library). 2D maps of *d*_P_ were then constructed for the trajectory frames. For each frame, the data for each lipid were mapped to two 512 × 512 point raster grids, one for each leaflet. To obtain the thickness, the raster data for the bottom leaflet was subtracted from the data for the top leaflet, resulting in a 512 × 512 raster grid containing the thickness data for each trajectory frame. The *d*_P_ values were then averaged over time. Area occupied by a lipid was calculated in a similar manner to thickness. First, the coordinates of each lipid in the membrane plane (the *XY* plane) were extracted from the trajectory. Then, using a modified version of lipyphilic’s AreaPerLipid functionality (which internally relies on the freud analysis library), the areas were calculated for each frame. This involved finding a Delaunay triangulation at points corresponding to the positions of the lipids’ phosphorus atoms. The dual of this triangulation was then computed, which gives a Voronoi diagram. The code then directly calculates areas of each cell and assigns it to the correct lipid. Subsequently, the 2D data was plotted onto 512 × 512 raster grids, one for each leaflet. The data was averaged in the same manner as for bilayer thickness. The order parameters (*S*_CD_) were calculated as previously described.^33^ A *k*-means clustering algorithm from the scikit-learn library was used to analyze data related to the release of styrene monomers.^34^ All images were prepared using VMD.^35^ Plots for the MD part were prepared using the matplotlib library.^36^

## Results

### Preparation and Characterization of PS Nanoplastic from Disposable Packaging

**Figure 1:**
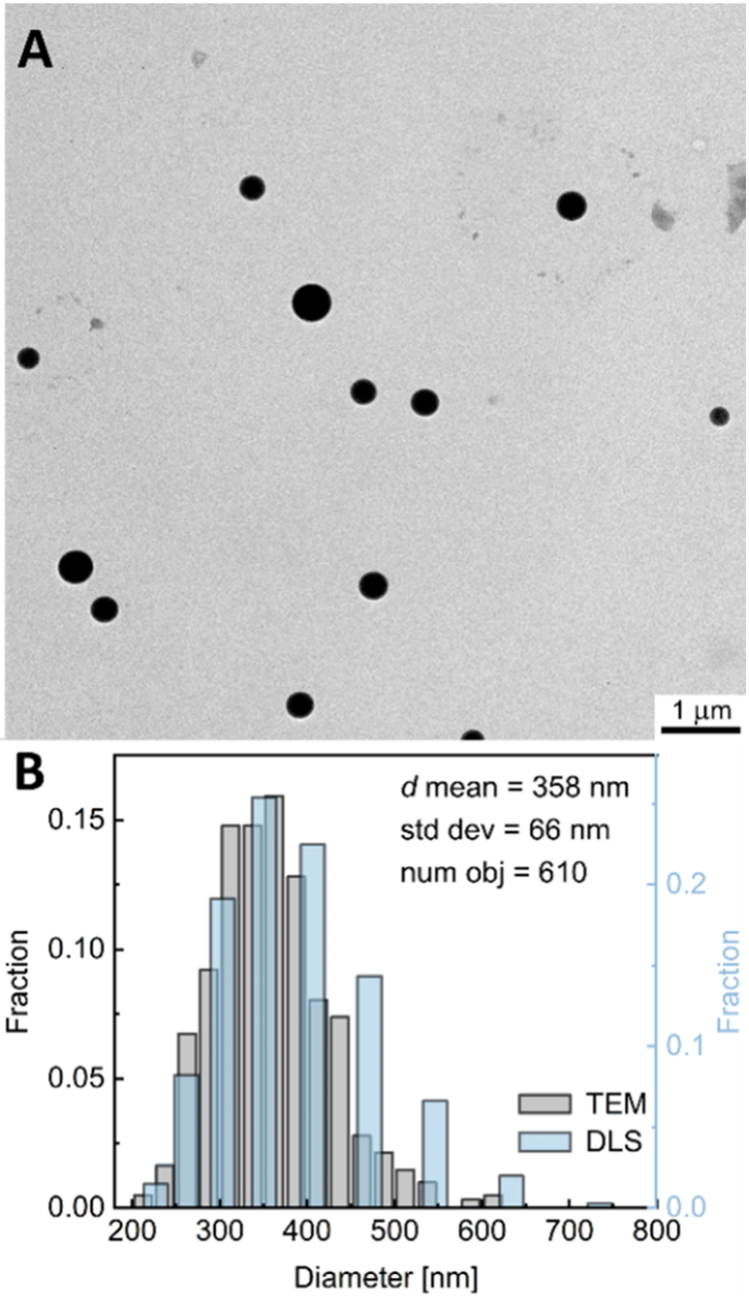
(A) TEM micrograph of the NR-stained PSNPs. (B) The distribution profile of particle diameters obtained from TEM imaging and DLS measurements.

PSNPs were prepared by dissolving the polystyrene material in THF and then precipitating in water. This procedure made it possible to encapsulate the fluorophore (NR) in the nanoparticles. The sizes of as-prepared particles were determined by DLS measurements (Figure 1). The hydrodynamic diameter (*d*_*z*_) of the PSNPs was 419 ± 2 nm in different preparation and polydispersity (PD) was lower than 0.1, indicating that the nanoparticles were homogeneous in size. Figure 1 shows a typical TEM image of the NR-stained PSNPs. The particles were spherical in shape with an average diameter of 358 ± 66 nm. The average size of PSNPs estimated from microscopic imaging is smaller than that determined from the DLS measurements. The discrepancy is likely due to the fact that TEM microscopy images particles in their dried state, while DLS measurements are made for hydrated objects (presence of a hydration shell on the surface of the nanoparticles). Another plausible explanation is the possibility of aggregation of PS nanoparticles. Similar observations were reported in previous studies. For example, Yu et al. found that the hydrodynamic diameter of unmodified PS NPs measured by DLS (103.5 ± 3.1 nm) was larger than the size (92.8 ± 6.9 nm) determined by scanning electron microscopy.

**Figure 2:**
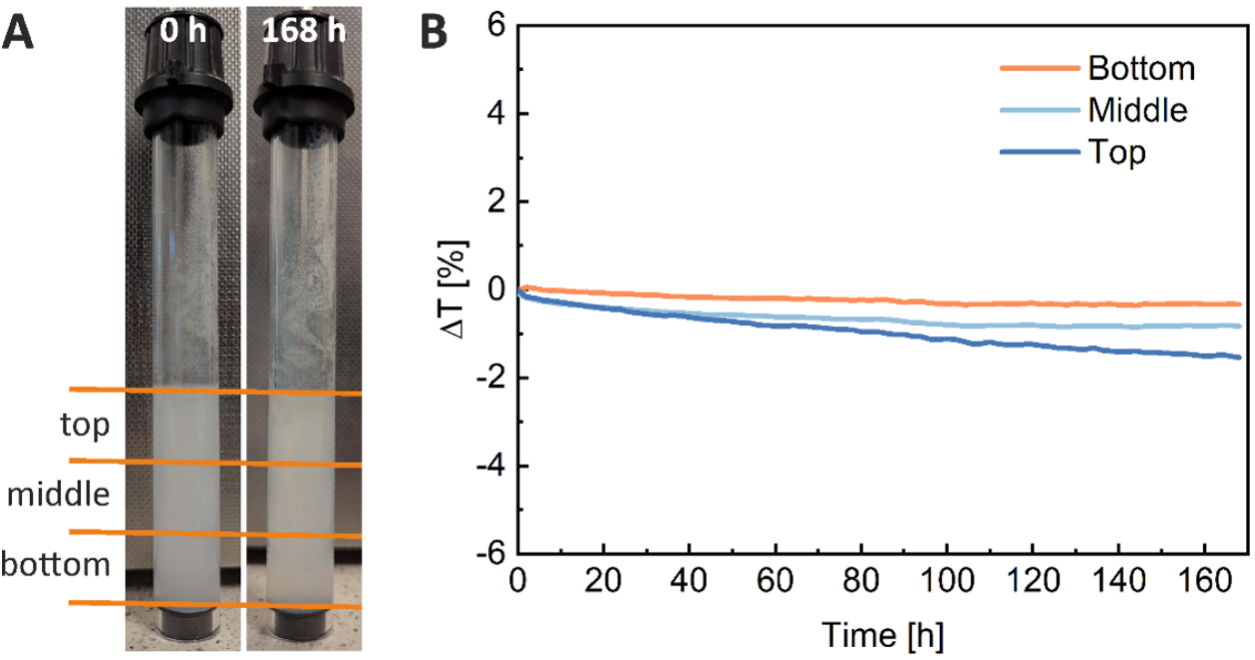
(A) Image showing the tube containing the PSNP dispersion before (0 h) and after Turbiscan measurements (168 h) and illustrating the division into top, middle, and bottom parts. (B) The mean changes in the transmittance of the PSNP dispersion (*c* = 1 mg/mL) over time in the top, middle, and bottom parts.

To evaluate the migration and aggregation of PS nanoparticles, changes in the transmittance (Δ*T*) of infrared light over time were measured in three parts of the sample (bottom, middle, and top) for a period of 128 h (Figure 2). A slight reduction in the transmittance of the dispersion was observed in all three parts of the sample. The decrease in transparency indicates an increase in the size of objects in the dispersion as a result of the nanoparticle aggregation phenomenon that occurs throughout the sample volume. However, the changes in the Δ*T* values of the sample are very negligible. For the upper part, the reduction is less than 1.5% after a period of 1 week, which shows that the PSNP dispersion is quite stable, and the aggregation is a marginal phenomenon. The high stability of the PS nanoparticle is also indicated by the zeta potential of PSNPs. The ζ -potential that depends on the surface charge of the nanoparticles is important for their stability in suspension and related to interactions between particles. Nanoparticles with zeta potentials less than −30 mV or greater than 30 mV are generally considered to be stable due to strong electrostatic repulsion preventing aggregation.^19^ The ζ value measured for PS nanoplastic prepared in this study was −30.35 mV, indicating that they carried sufficient negative charges to prevent their aggregation.

### Interactions of PSNPs with Lipid Membranes

**Figure 3:**
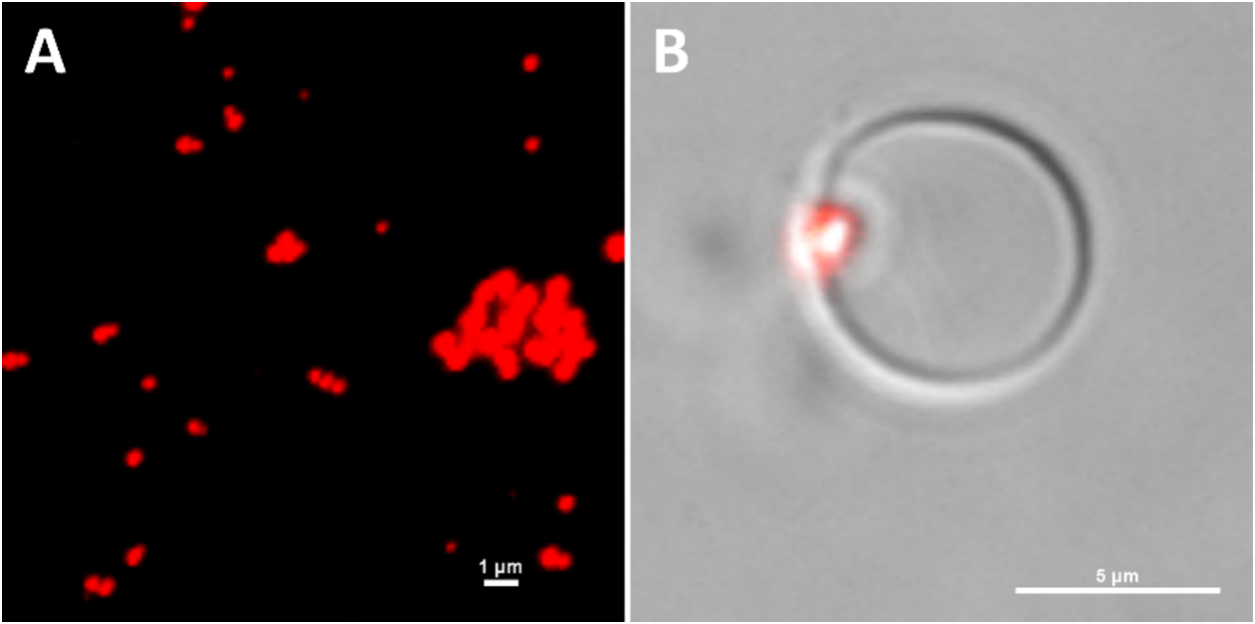
Confocal micrographs of NR-stained PSNPs dispersed in PBS (A) and POPC GUVs incubated with NR-stained PSNPs superimposed on the transmitted light image of the sample (B). NR fluorescence is shown in red.

We used laser scanning confocal microscopy (LSCM) to directly observe the interaction of PS nanoplastic with the lipid membrane. For this purpose, PSNPs were stained with NR at the stage of their preparation. The dispersion of NR-stained PSNPs was then imaged under the confocal microscope to confirm the confinement of the fluorophore in the nanoparticles. Admittedly, the size of PSNPs is small, but as shown in Figure 3A, the light emitted by these particles can be detected by the confocal system. GUVs were then incubated with the NR-stained PSNPs overnight and observed under the confocal microscope. Several events of PSNP interaction with the POPC membrane were observed during the experiment (Figure 3B). However, the resolution of confocal microscopy is too low to assess whether there is membrane penetration by PSNPs or only their adsorption on the surface of the bilayer. The penetration of PS particles of this size into the lipid membrane seems to be hindered, which is consistent with the results of MD simulations, showing that the affinity of PS nanoplastics for lipid membranes decreases with increasing nanoparticle size.^6^ Nevertheless, the microscopic experiment clearly shows that polystyrene nanoplastic can adsorb on or enter the zwitterionic membrane.

### Miscibility of PS with the POPC Matrix

**Figure 4:**
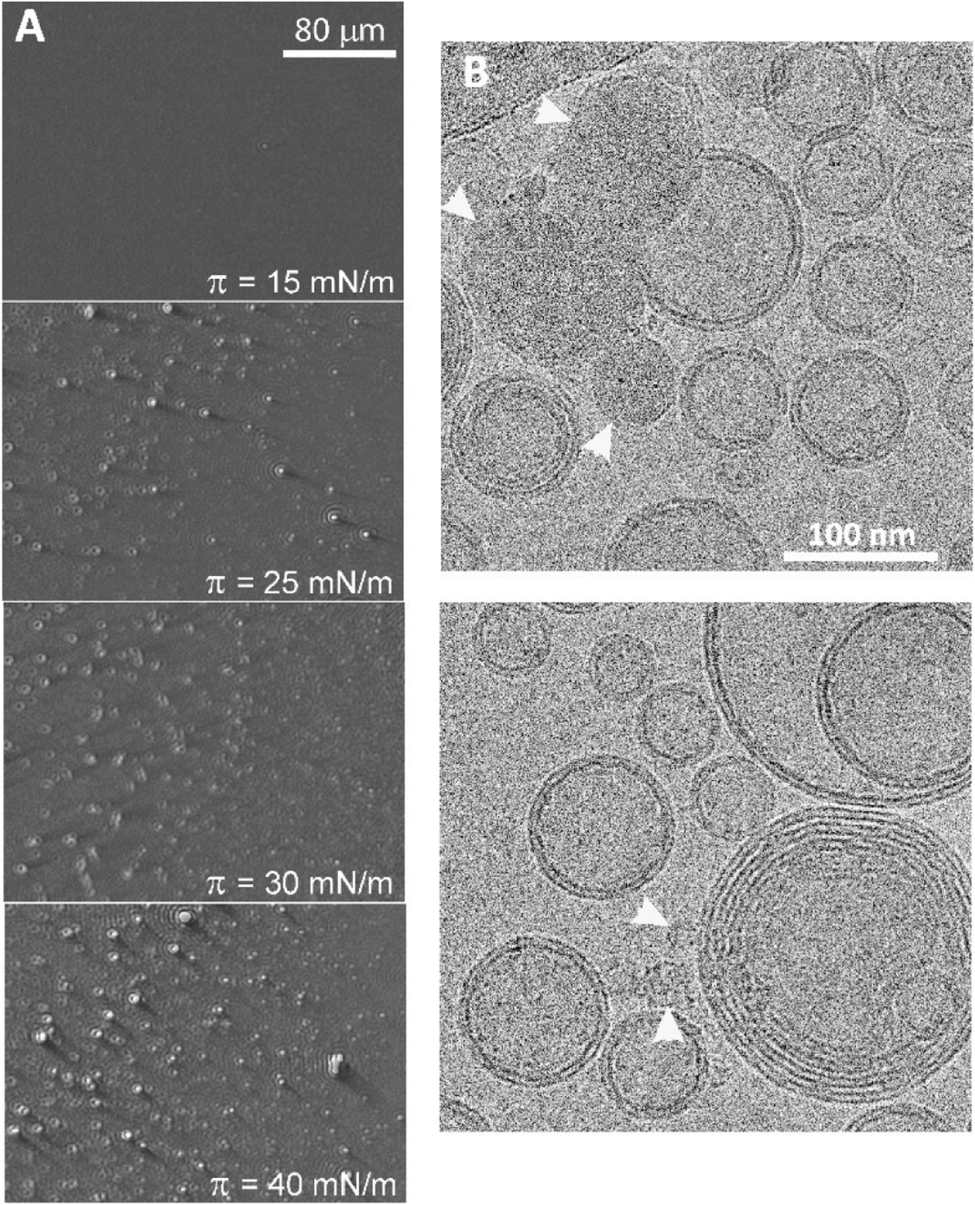
(A) Brewster angle microscopy (BAM) images taken at different stages of POPC/PS (5 wt %) film compression at 25 °C. (B) Cryo-EM micrographs of POPC liposomes and PS nanoparticles formed during rehydration of PS–POPC films. White arrowheads indicate PS nanoparticles.

The miscibility of PS with the POPC matrix was investigated by BAM microscopy, which enables visualization of the morphology of ultrathin films deposited at the air/water interface. The presence of a film causes partial reflection of the *p*-polarized light incident at the Brewster’s angle, and its intensity is determined by the film’s refractive index and thickness. Figure 4A shows BAM images recorded during compression of a POPC monolayer containing 5 wt % of PS. For pure POPC, a uniform morphology was observed, indicating that the monolayer is tightly packed and relatively homogeneous.^37^ In the case of the PS-doped POPC monolayer, domains of increased brightness appeared during compression, indicating the formation of structures of greater thickness when the surface pressure is increased above 15 mN/m. Their size and number gradually increase with the compression of the POPC/PS film. The properties of the monolayer at surface pressures of about 30 mN/m were shown to correspond to those of the bilayer.^38^ Therefore, the presence of bright spots in the BAM picture of the monolayer at this surface pressure indicates limited mutual miscibility of PS chains in the POPC bilayer.

To study the miscibility of PS with POPC matrix at the nanometer scale, we performed cryo-EM morphology imaging. Taking advantage of the fact that both lipid and polystyrene are soluble in chloroform, a dry lipid film was prepared with an admixture of PS (5 wt %), which was then rehydrated. The objects formed during rehydration were imaged under a cryo-EM microscope (Figure 4B). The micrographs reveal that the rehydration of the mixed polymer–lipid films led to the formation of predominantly unilamellar liposomes with a distinct POPC bilayer structure and the creation of separate PS nanoparticles of different sizes either embedded in the POPC bilayer or released into the aqueous phase. This is probably due to the high stability of PS nanoparticles in the aqueous phase, as demonstrated by the Turbiscan experiments. These results indicate that polystyrene exhibits very limited solubility in the hydrophobic region of the POPC membrane on the nanometer scale as well.

### Unbiased Simulations of PSNP–Lipid Bilayer Interactions

**Figure 5:**
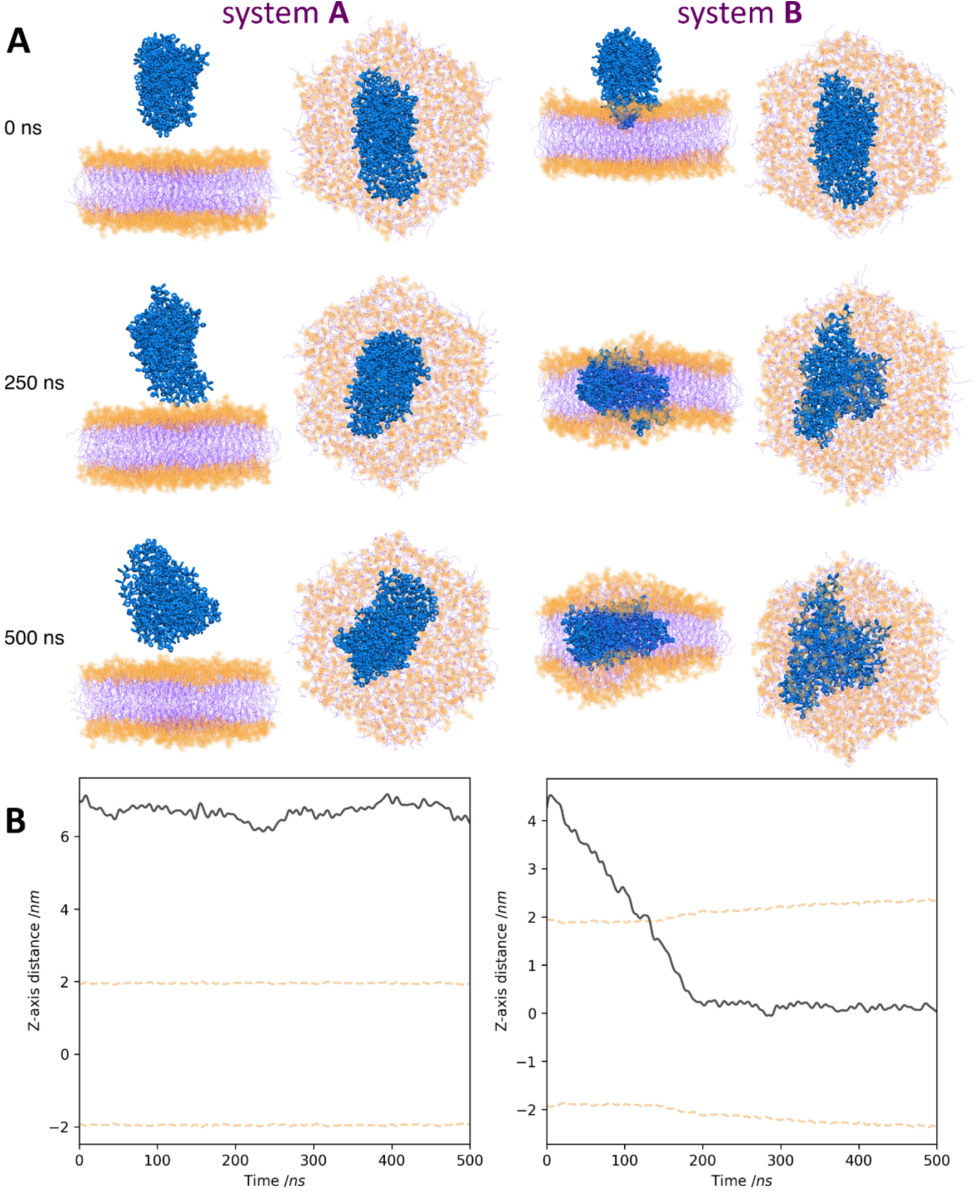
(A) Snapshots showing from the side and top the configuration of systems **A** and **B** after different simulation times. The PS aggregate is shown in blue. POPC headgroups and acyl groups are depicted as transparent orange spheres and transparent purple lines, respectively. Water and ions are omitted for the sake of clarity. (B) Trajectories of the center of mass of PSNP for systems **A** and **B**. Horizontal dashed lines indicate the average position of the phosphorus atoms in two membrane leaflets.

Microsecond-time scale AA MD simulations of systems containing the POPC membrane and the PS nanoparticle mimicking nanoplastic were performed to study the PSNP-membrane interaction on the molecular level. Polymer nanoparticles were constructed from 14 PS chains that formed a compact coil with irregular shapes during simulation. The polymer aggregate was homogeneous up to a distance of about 2 nm from its center of mass. Its mass density was about 1.026 g/cm^3^, and the size of its long axis was about 8 nm. The value of the mass density is very close to that determined experimentally (1.040–1.065 g/cm^3^) for polystyrene beads.^39^ Therefore, the PSNP can serve as a good model of PS nanoplastics. The PSNP was placed close to (system **A**), partially immersed in (system **B**), or completely embedded in (system **C**) the zwitterionic membrane. Snapshots showing the evolution of the simulated system over time are depicted in Figures 5A and S1. Figure 5B shows how the center of mass (COM) of PSNP moved along the bilayer normal during the simulations. In the case of system **A**, the PS nanoparticle resided near the polar region of the membrane but showed no tendency to penetrate the membrane. We did not observe spontaneous entry of the PSNP into the bilayer, even after 500 ns of simulation. The energy barrier of the polar region of the membrane seems to be too high for PS nanoparticles to overcome it on such time scales. In contrast, the PSNP that was slightly inserted into the lipid bilayer (system **B**), effectively moved to its center. Within ca. 180 ns, the whole NP was submerged in the lipid membrane and remained there for the rest of the simulation time, causing an increase in the POPC membrane thickness (Figure 5). Once the nanoparticle had fully entered the hydrophobic region of the membrane, the lipids rearranged quickly (within 200 ns) to cover and isolate PSNP from the solvent.

In the case of system **C**, the nanoparticle was initially placed with its long axis parallel to the normal bilayer, piercing the bilayer (Figure S1). The system was characterized by a more symmetric and relaxed bilayer at the beginning of the simulation. During the simulations, the aggregate changed its orientation to avoid contact with water. Within about 300 ns, the elongated PSNP laid in the bilayer with its long axis parallel to the membrane surface and was completely surrounded by the POPC molecules. This qualitatively shows that covering the nanoparticles with lipids is more energetically favorable to the system than contacting the aggregate with water.

### Behavior of PS Coil in Aqueous Phase and in Lipid Bilayer

**Figure 6:**
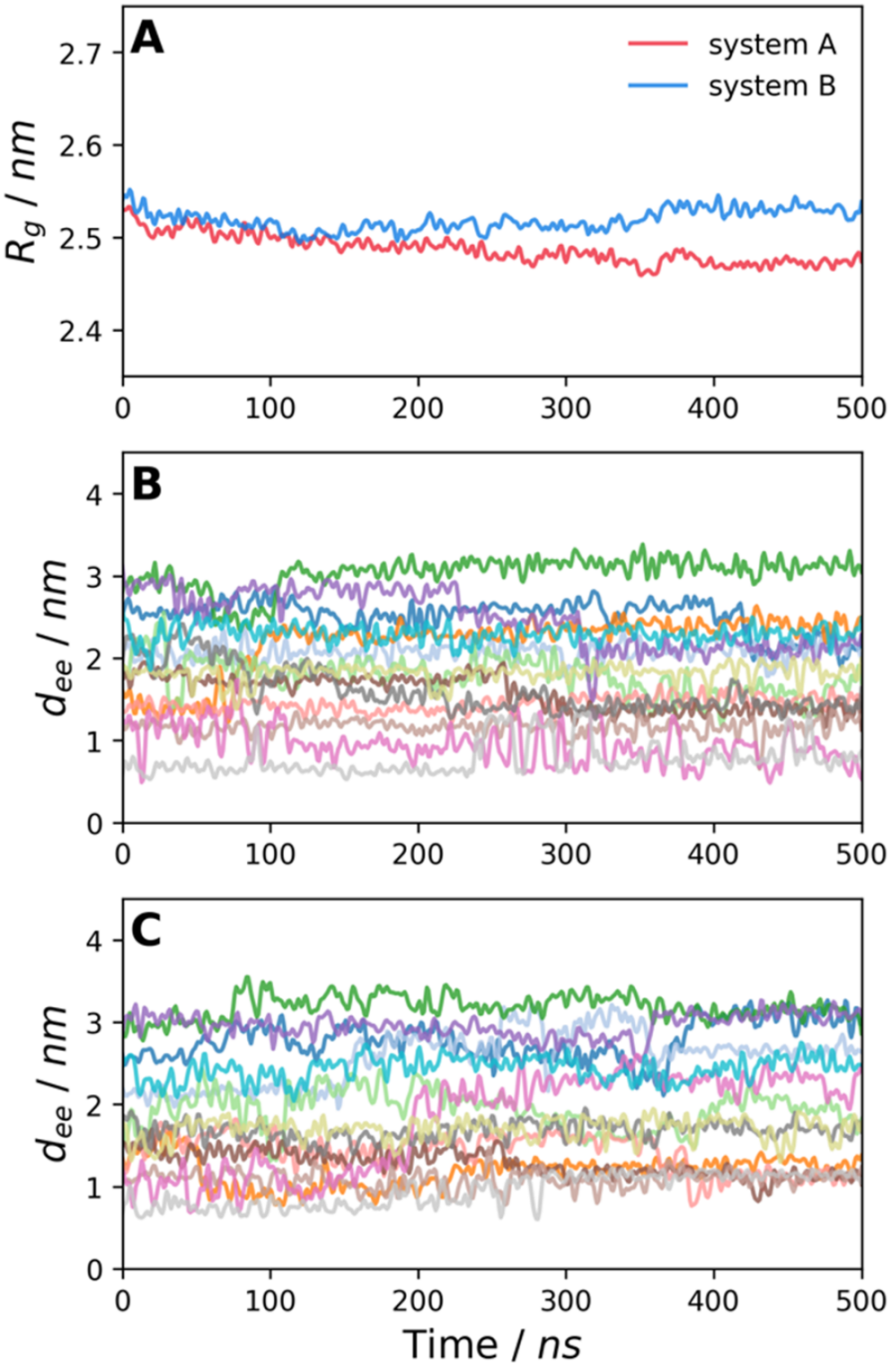
Radii of gyration (*R*_g_) of the PS nanoparticle (A) and end-to-end distances (*d*_ee_) of individual PS chains for systems **A** and **B** (B, C). Each color represents a different chain.

To investigate changes in the structure of PSNP located in the aqueous phase and in the membrane, we analyzed the radii of gyration (*R*_g_) of the PS coil and the end-to-end distances (*d*_ee_) of the polymer chains for systems **A** and **B** (Figure 6). In the case of system **B**, the *R*_g_ value was almost constant during the entire simulations, showing that the structure of the PS nanoparticle does not change after entering the membrane. In contrast, for system **A**, the gyration radius decreased slightly from 2.55 to 2.48 nm, suggesting that the coil became more compact. However, we believe that this change is due to the irregular shape of the coil, where even small conformational changes of the PS chains on the surface can largely affect the *R*_g_ value of the whole aggregate. Furthermore, the end-to-end distances of the individual oligomer chains in the nanoparticles changed only slightly over time (Figure 6B,**C**), showing that the PS coils located in the aqueous phase as well as in the POPC bilayer did not disaggregate.

### Effect of PS Nanoparticles on the Molecular Organization of the POPC Bilayer

**Figure 7:**
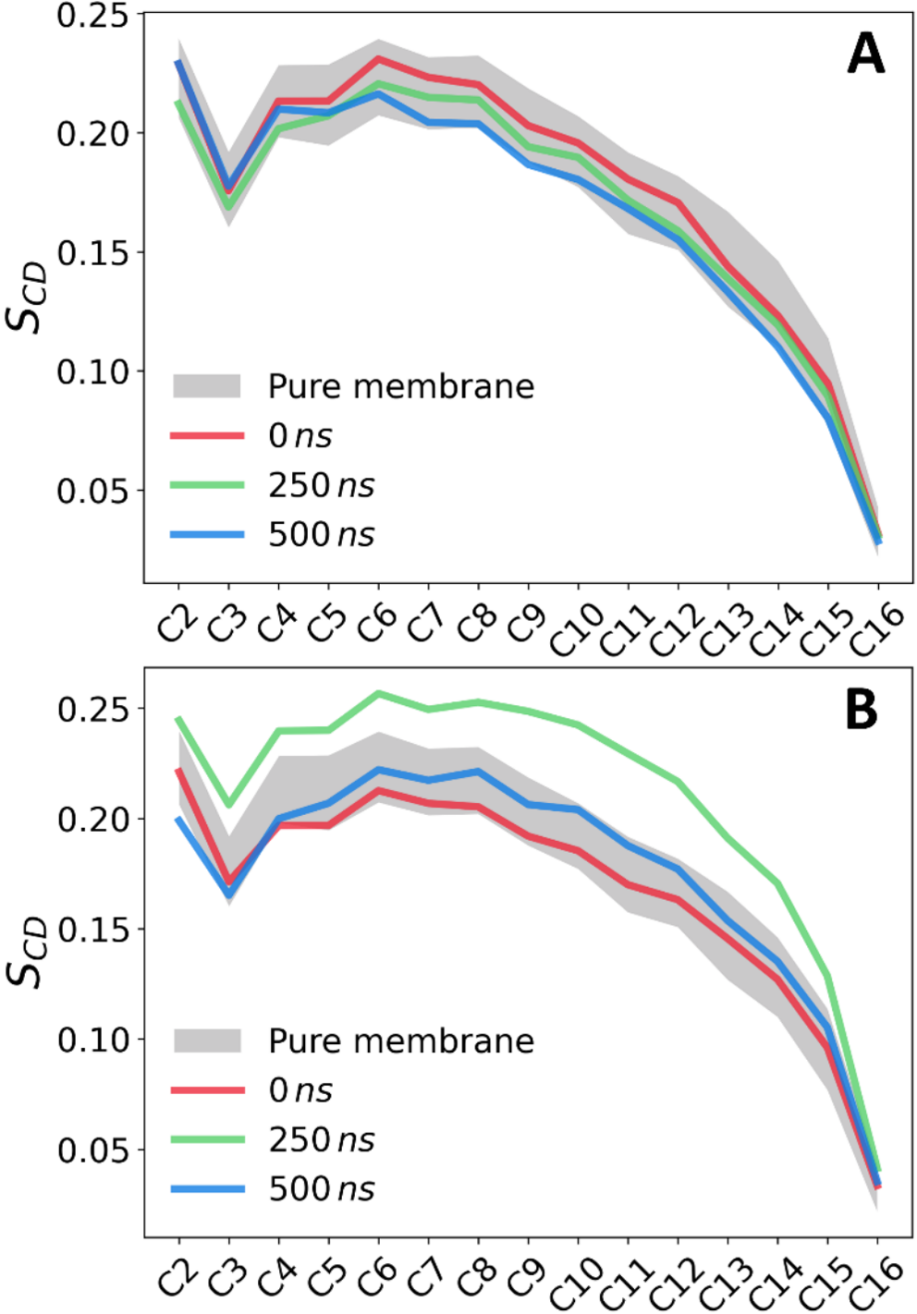
Acyl chain deuterium order parameters (|*S*_CD_|) for systems A and B after different simulation times. The mean |*S*_CD_| and its standard deviation for the pure POPC bilayer are shown as an orange-shaded area.

We then focused on the effect of PS nanoparticles on the properties of the zwitterionic bilayer. To estimate the extent of PSNP-induced membrane disturbance, area per lipid (APL), membrane thickness (*d*_P_), and order parameter (*S*_CD_) of lipid molecules were calculated (Table 2 and Figure 7). These values were compared with those for the pure POPC membrane as a reference system. For system **A**, in which no significant interaction of PSNP with the membrane was observed, the calculated values were comparable with those for the pure POPC membrane. The observed changes in *S*_CD_ values (Figure 7A), are within the range of changes for pure POPC membrane and are therefore the result of natural membrane fluctuation. In the case of system **B**, a marked increase in membrane ordering is evident at 250 ns (Figure 7B). This is the point in time at which the aggregate has just penetrated the center of the bilayer (Figure 5). The curve for the average deuterium order parameter shows the high ordering of the lipids in the bilayer. Over time, the bilayer reorganized to absorb the nanoparticle, and the ordering of the lipid chains decreased, but the *S*_CD_ parameters remained at the upper edge of the natural fluctuations of the undisturbed membrane, indicating that the presence of PS nanoplastics in the membrane forced the partial ordering of the membrane. This is more apparent in the behavior of system **C**, where the *S*_CD_ values exceed the upper edge of the natural fluctuations compared to the pure bilayer (Figure S2). As time passes, the membrane becomes more ordered, especially in the C8–C14 region.

**Table 2:** Averaged Values of Area Per Lipid (APL) in the Top and Bottom Leaflets and Membrane Thickness (*d*_P_)^a^^b^

system	APL top [nm^2^]	APL bottom [nm^2^]	APL [nm^2^]	*d*_P_ [nm]
**POPC**	0.64 ± 0.13	0.64 ± 0.13	0.64 ± 0.13	3.93 ± 0.54
**A**	0.64 ± 0.10	0.65 ± 0.12	0.65 ± 0.11	3.89 ± 0.54
**B**	0.64 ± 0.10	0.67 ± 0.11	0.65 ± 0.10	4.67 ± 1.39
**C**	0.66 ± 0.19	0.63 ± 0.13	0.64 ± 0.16	4.85 ± 1.41

a Data were averaged over the last 50 ns of the simulation.

b Defined as an average distance between the phosphorus atoms in the opposite leaflets.

The presence of large PS aggregates (roughly 4 nm in size in the bilayer-normal direction) in the lipid membrane results in an increase in its curvature, visible in the snapshots (Figures 5A and S1). To illustrate the distribution of system components along the normal to the bilayer, we calculated the mass density across the membrane of selected lipid groups, PS, and water (Figure S3). For systems B and C, the final linear density distribution across the two bilayers is remarkably similar, indicating that this is a common final state for membranes with incorporated PS particles of this size. PSNP is located preferentially inside the POPC bilayer. Its distribution shows a single maximum at about 0 nm from the center of the membrane. The entire bilayer is also significantly broadened, which is most evident in the distribution of the hydrophilic parts of the bilayer. Instead of a monomodal distribution (per leaflet), a pronounced bimodal distribution is observed, due to the lipid layer covering the nanoparticle, which creates two thickness domains. The presence of PSNP has a striking effect on the thickness of the bilayer, effectively doubling it at the thickest point.

**Figure 8:**
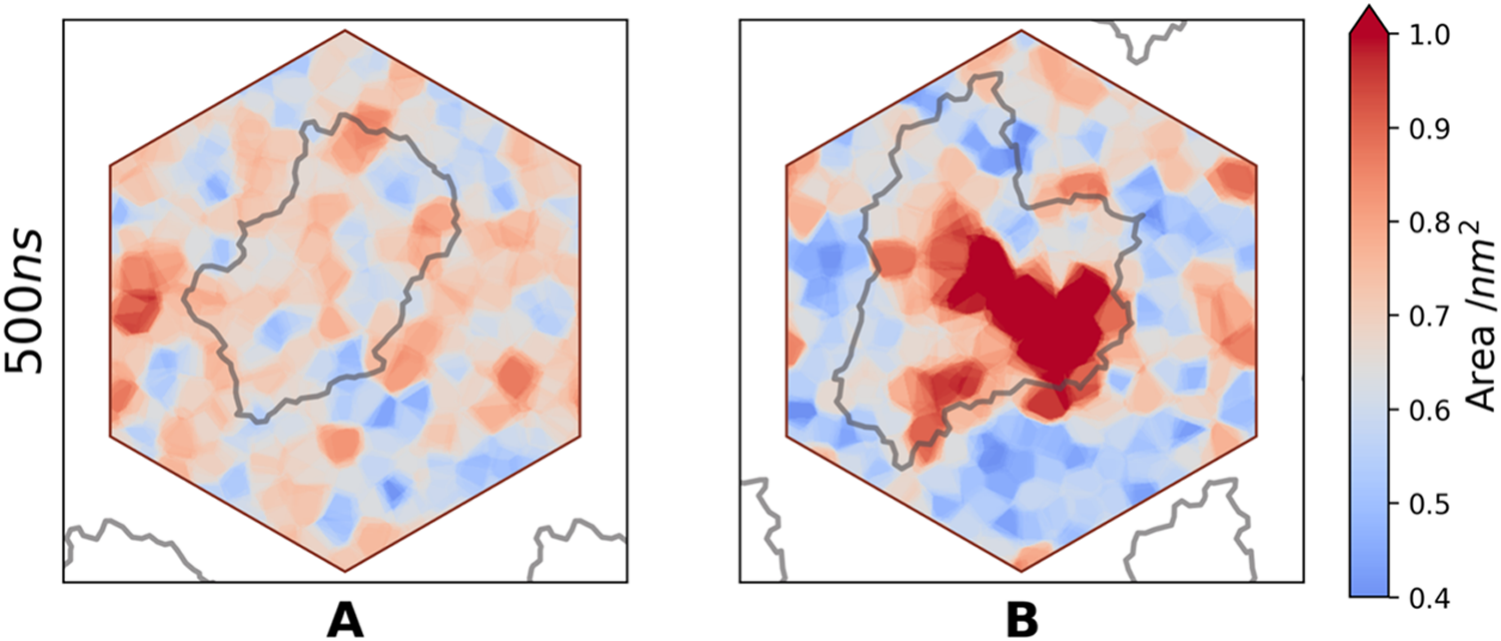
2D maps of the area per lipid (APL) for systems **A** and **B**. The maps were calculated by averaging the data over a 10 ns window at the end of the trajectory. The PS coil and its periodic images are projected onto the membrane surface and shown as a gray outline overlaid on the map. The color scale is centered at 0.64 nm^2^, which corresponds to average APL of the POPC membrane. Unit cell boundaries are marked in burgundy.

As PSNP did not disaggregate when immersed in the membrane, we calculated two-dimensional APL maps to analyze the local perturbation of the POPC bilayer properties induced by the PS nanoplastic (Figures 8 and S4). For system **A** (PSNP was outside the membrane, Figure 5A), the map shows only natural fluctuations in the bilayer. This is in stark contrast to the maps for systems **B** and **C**, where the aggregate gradually dipped into the interleaflet region and was covered with lipid molecules as the simulations progressed. The presence of PSNP has a clear effect on the area occupied by the lipid at the end of the simulations. Lipids covering the aggregate are characterized by markedly lower packing, which is evident as an increase in APL in the area enclosed by the nanoparticle contour. Interestingly, lipids that are not in direct contact with the aggregate appear slightly compressed.

### Release of Styrene from the PSNP

**Figure 9:**
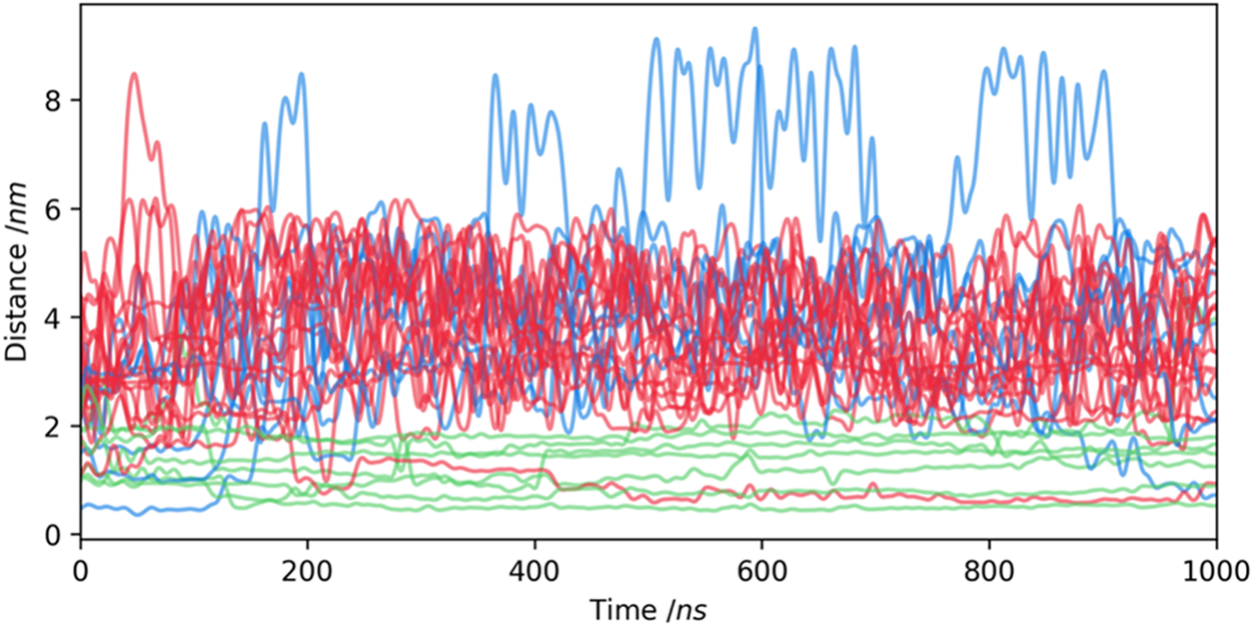
Time profiles of the distance between the centers of mass of PSNP and each STR molecule. Distances were calculated using the MDAnalysis library built-in periodic distance calculation utilities. The trajectories of each styrene molecule were clustered using k-means (with *k* = 3 clusters) clustering algorithm and each was assigned to a category: immobile (green), released (red), and escaped (blue).

Polystyrene nanoplastic may be contaminated with styrene (as a result of the presence of unreacted monomer)^10^ or the biodegradation of PS particles and the release of styrene molecules.^40,41^ To investigate the process of possible STYR release from PS nanoplastic into lipid membranes, we designed system **C**, in which 32 STYR molecules were enclosed in the PSNP. The STYR-laden nanoparticle was then immersed in the POPC bilayer. Figure 9 shows how the distance of the STYR molecules from the center of mass of the PS coil changed during the simulations. Due to the irregular shape of the PS nanoparticle, interpretation of the results was difficult. Therefore, we used the *k*-means clustering algorithm to divide the STYR molecules into three groups: immobile (confined inside the nanoparticle), released (located peripherally and released into the bilayer), and escaped (moving from the membrane to the aqueous phase). Clustering was performed using the standard deviation of the distance between COM of PSNP and the STYR molecule.

During the simulation, desorption of styrene molecules located close to the surface of the aggregate occurred, even before the aggregate completely penetrated the membrane. As the simulation progressed, more encapsulated STYR was released. The monomer molecules moved freely inside the bilayer and therefore their distance from the COM of the aggregate changed dynamically. A small number of STYR molecules were capable of spontaneously passing into the aqueous phase and re-entering the membrane. For 6 monomer molecules, it was observed that their distance from the center of the nanoparticle remained almost constant. These molecules were trapped inside the PSNP, so their movements were blocked. It should be noted that as a consequence of its irregular shape and size, PSNP had a well-developed surface area relative to its volume. Therefore, most of the monomer molecules were located close to the nanoparticle surface, leading to the release of 18 pollutant molecules. Our observations indicate that small-molecule additives in nanoplastics, such as unreacted monomers are released into lipid membranes if they are located close to the particle surface, whereas molecules present in the core are not released in 1 μs time scales.

## Discussion

*In vitro* studies clearly show that PS nanoplastics are effectively internalized by various types of cells.^42,43^ The pathways of cellular uptake of micro/nanoplastics ranging in size from tens of nanometers to micrometers can be divided into two main ways: active transport and passive diffusion.^44^ Active transport involves an endocytosis mechanism that usually occurs during the cellular uptake of relatively large particles. Endocytosis is considered as the key mechanism of particle uptake by cells.^45^ Particles that are internalized by cells in this way can form aggregates that are wrapped by cell membranes and transported into cells.^46^ Passive transport, which typically occurs during cellular uptake of small particles, involves diffusion across the cell membrane and release into the cell cytoplasm, allowing nanoparticles to enter the cell directly. Experimental studies have shown that PSNPs are able to enter mammalian cells through passive translocation.^40^ The mechanism of passive penetration of a plastic nanoparticle into a cell requires overcoming the barrier that is the plasma membrane. Therefore, learning about the initial interactions of the nanoparticle with the lipid membrane used as model cell membranes can aid in understanding the passive diffusion of nanoplastics into cells. Here, we examined the interactions between the PS nanoplastic and the zwitterionic membrane.

### Penetration of PSNP into the Lipid Membrane

The surface charge and hydration of nanoparticles are substantial factors that can affect their entry into lipid membranes. Our ζ-potential measurements indicated that nanoparticles made of PS waste carry a net negative charge on their surface. The presence of a negative surface charge results in the formation of an electrical double layer due to the increased concentration of counterions close to the nanoparticle surface and the hydration surrounding the particles. Nevertheless, the magnitude of the potential measured at the shear plane indicates a high stability of the PSNP dispersion resulting from the mutual repulsion of the nanoparticles. The colloidal stability of PSNP was confirmed by turbidimetric measurements. Negative values of the zeta potential for nanoparticles made of pure PS were reported previously. Yu et al. showed that the ζ value of PS nanoparticles depends of pH and decreased from ca. −33 to −40 mV with increasing pH from 3 to 6.^47^ A slightly lower zeta potential (ca. −43 mV) was found for PS nanoparticles with a diameter of about 33 nm dispersed in ultrapure water.^48^ However, the degree of hydration of PSNPs has not been studied experimentally.

Using confocal microscopy, we confirmed that despite their negative surface charge and hydration, PSNPs can interact with the zwitterionic membrane (adsorbing onto or incorporating into it). However, since only a few cases of interaction of the lipid membrane with PSNPs were observed even after long incubation times, it can be assumed that the adsorption/entry of PS particles of this size into the lipid membrane is hindered. As shown previously, the POPC membrane is also negatively charged, as indicated by zeta potential measurements (the ζ potential of POPC liposomes ranged from −4 to −12 mV)^49^ which, combined with the large negative charge on the surface of PSNPs, contributes to the repulsion between PS nanoplastics and lipid membranes. The results of our MD simulations are consistent with these microscopic observations.

The simulation of system **A**, in which PSNP was initially placed in the aqueous phase, revealed that the nanoparticle exhibited no tendency to penetrate the POPC membrane. It should be noticed that the POPC headgroups are strongly hydrated, which may act as an effective barrier to penetration of PSNPs into the carbohydrate region of the membrane. Our previous calculations showed that the headgroups of POPC membrane are hydrated by about 11 water molecules per N(CH_3_)_3_^+^ lipid group.^50^ The entry of a nanoparticle made of a hydrophobic material into the membrane would therefore require the removal of hydration water in the NP-bilayer contact area. As noted previously, the energy cost of removing hydration water is much higher than the weak interaction between headgroups and hydrophobic polymer.^10^ For this reason, PSNPs are inclined to remain in the aqueous phase if they are initially placed there. The small immersion of PSNP into the polar region of the bilayer (system **B**) means that the barrier associated with membrane hydration is overcome, and the nanoparticle spontaneously penetrates the membrane in a short time. The mass density profiles (Figure S3) demonstrate that the nanoparticles located preferentially in the center of the bilayer.

### Behavior of PSNPs in Lipid Membranes

Our simulations (systems **B** and **C**) clearly revealed that PSNPs did not disaggregate when embedded in the POPC bilayer. This result is consistent with the physicochemical properties of polystyrene, which is in the solid (glassy) state at room temperature, i.e., the mobility of the polymer chains is severely limited. Polymer particles become liquid when heated above the so-called glass transition temperature (*T*_g_). The *T*_g_ value for PS strongly depends on the molecular weight of the polymer. For the molecular weights of the PS chains used in our simulations, it is approximately 78 °C, well above the temperature of the simulated systems.^51^

The miscibility of PS and POPC was investigated by BAM and cryo-EM microscopy. Taking advantage of the fact that both POPC and PS are soluble in chloroform, we prepared mixed films on the water subphase. BAM images recorded during compression of the mixed POPC/PS films demonstrated phase separation of the polymer from the lipid matrix and the formation of PS domains. Also, cryo-EM imaging demonstrated the phase separation of PS from the hydrophobic region of the POPC membrane. In this case, the formation of PS nanoparticles was observed, which were embedded in the membrane or released into the aqueous phase. Our BAM and cryo-EM observations are consistent with the results of studies on the polymerization of styrene solubilized in a bilayer of vesicles made of dioctadecyldimethylammonium bromide (DODAB)^52^ or dimiristoylphosphatidylcholine (DMPC)^53^ vehicles. Using cryo-EM microscopy, it was shown that due to the complete phase separation between the produced polystyrene and the vesicle bilayer, polymerization of styrene in DODAB vesicles (with C18 chains) leads to morphologies in which the polymer particles were encapsulated in the lipid bilayer. In the case of polymerization of styrene solubilized in DMPC liposomes (with C14 chains), mainly free PS nanoparticles were formed, and less frequently the particles encapsulated in the lipid bilayer were observed. The authors concluded that the more fluid and thinner DMPC bilayer is unable to pertain the polymer particle in its interior and therefore releases the beads after exceeding a certain limiting size.^30^

It should be noted that although for the purposes of this study we obtained PSNPs from EPS from take-out packaging certified for contact with food, which may end up as waste in the environment, the preparation method used allowed us to obtain the nanoparticles with a spherical shape (Figure 1). In addition, the resulting PSNP sizes were quite narrow and optimized for confocal microscope imaging. PS nanoplastics formed in the environment as a result of PS waste degradation processes are characterized by irregular shapes and a wide size distribution. As shown previously, both the shape and size of the plastic particles can affect the interaction with cell membranes^54^ and cellular uptake.^55^ Therefore, further research should focus on nanoparticles with irregular shapes and a wider size distribution.

## Conclusions

We performed an analysis of the interaction of PS nanoplastics with zwitterionic lipid membranes (used as a nonprotein model for cell membranes) using CLSM, BAM, and cryo-EM observations combined with all-atom MD simulations to gain insights relevant to understanding the passive diffusion of nanoparticles into cells. Our results indicate that the penetration of PS nanoplastic obtained from disposable food packaging into zwitterionic membranes is hindered. First, such nanoparticles are hydrated and have a high negative surface charge when dispersed in aqueous media. Also, the surface of the POPC bilayer is highly hydrated, so the penetration of PSNPs into the zwitterionic membrane requires the energetically expensive removal of water molecules at the interface between the nanoparticles and the lipid membrane, which is an effective barrier to the penetration of PSNPs into its hydrophobic region. In addition, the POPC membrane is negatively charged, which, combined with the high negative charge on the surface of PSNPs, contributes to the repulsion between PS nanoplastics and lipid membranes. However, overcoming this energy barrier by slightly inserting the PS nanoparticle into the polar region of the membrane leads to its rapid entry into the center of the bilayer and coating its surface with lipid molecules.

Another important outcome of our study is that PS nanoplastics do not disaggregate after penetrating the lipid membrane, which affects the molecular structure of the bilayer. The presence of large PS aggregates in the lipid membrane causes a significant increase in its curvature, and the lipid layer covering the nanoparticle is characterized by noticeably lower packing.

It is important to note that PS nanoplastics introduced into biological media become coated with proteins present there, resulting in the formation of a coating on their surface known as a protein corona. The presence of this coating has a critical effect on the interaction of nanoparticles with cells.^56^ Therefore, we believe that the mechanism of passive uptake of PS nanoplastics is more complicated than simple partitioning of the nanoparticles into the lipid membrane and may be related to the presence of proteins on the surface of the cell membrane, which merits further research.

## Supporting Information Available

Snapshots of system **C**; trajectory of the center of mass of PSNP for system **C;** acyl chain deuterium order parameters for system **C**; mass density profiles; and 2D maps of the area per lipid for system **C** (PDF)
