# Supplementary material for: Interaction of Polystyrene Nanoplastic with Lipid Membranes
Source: J Phys Chem B. 2025 Apr 10;129(16):4110–22. doi: 10.1021/acs.jpcb.5c00738 (PMC12035802; doi:10.1021/acs.jpcb.5c00738)
Supplement: Supplementary file 1 — jp5c00738_si_001.pdf [file jp5c00738_si_001.pdf]

Interaction of Polystyrene Nanoplastic with Lipid Membranes

Grzegorz Łazarski,[†,‡] Natan Rajtar,[†,‡] Marek Romek,[§] Dorota Jamróz,[†] Michał Rawski,[$] and Mariusz Kepczynski[†,*]

[†] Jagiellonian University, Faculty of Chemistry, Gronostajowa 2, 30-387 Kraków, Poland

[‡] Doctoral School of Exact and Natural Sciences, Jagiellonian University, Prof. S. Łojasiewicza 11, 30-348 Krakow, Poland

[§] Department of Cell Biology and Imaging, Institute of Zoology and Biomedical Research, Jagiellonian University, 9 Gronostajowa Street, 30-387 Kraków, Poland

[$] National Synchrotron Radiation Centre SOLARIS, Jagiellonian University, 98 Czerwone Maki Street, 30-392 Kraków, Poland

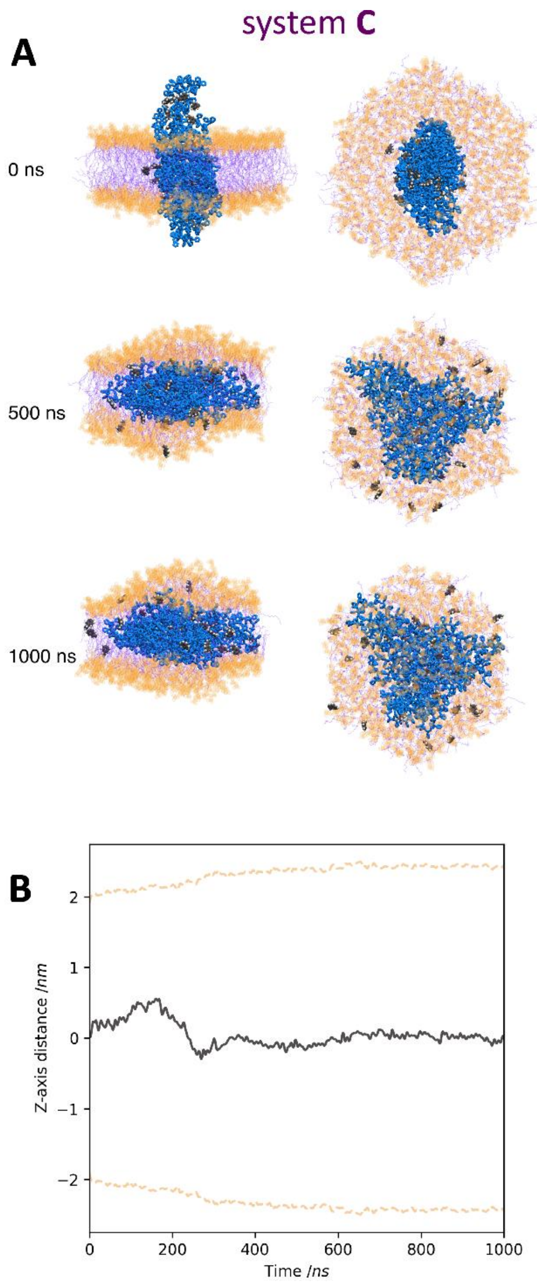

Figure S1. 1 (A) Snapshots of system **C** showing from the side and top the configuration after different simulation times. The PS aggregate is shown in blue. POPC headgroups are depicted as transparent orange spheres, and acyl groups are shown as transparent purple lines. Styrene monomers are shown as black and white spheres. Water and ions are omitted for clarity. (B) Trajectory of the center of mass of PS_NP for system **C.** Horizontal dashed lines indicate the average position of the phosphorus atoms in the two membrane leaflets,

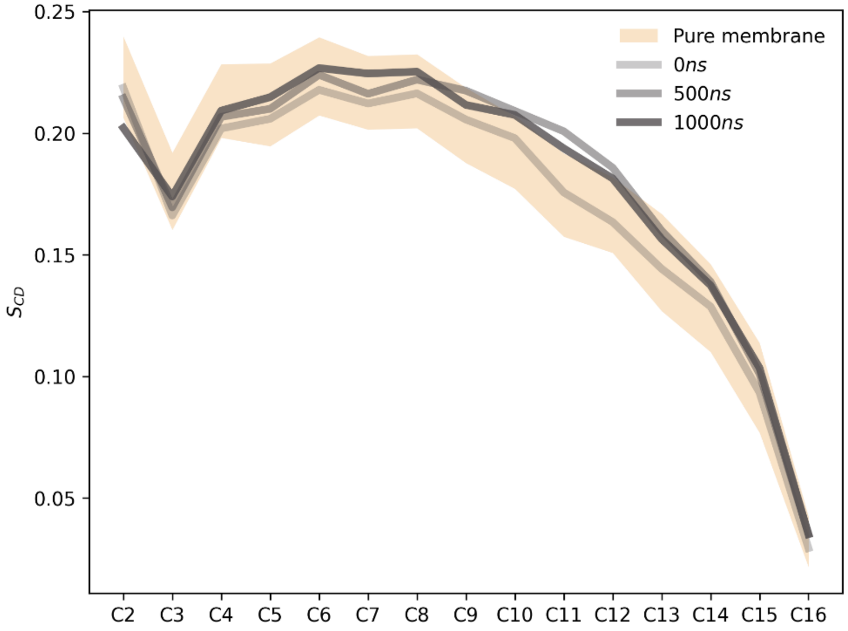

Figure S2. Acyl chain deuterium order parameters ($|S_{CD}|$) for system **C** after different simulation times. The mean $|S_{CD}|$ and its standard deviation for the pure POPC bilayer are shown as an orange shaded area.

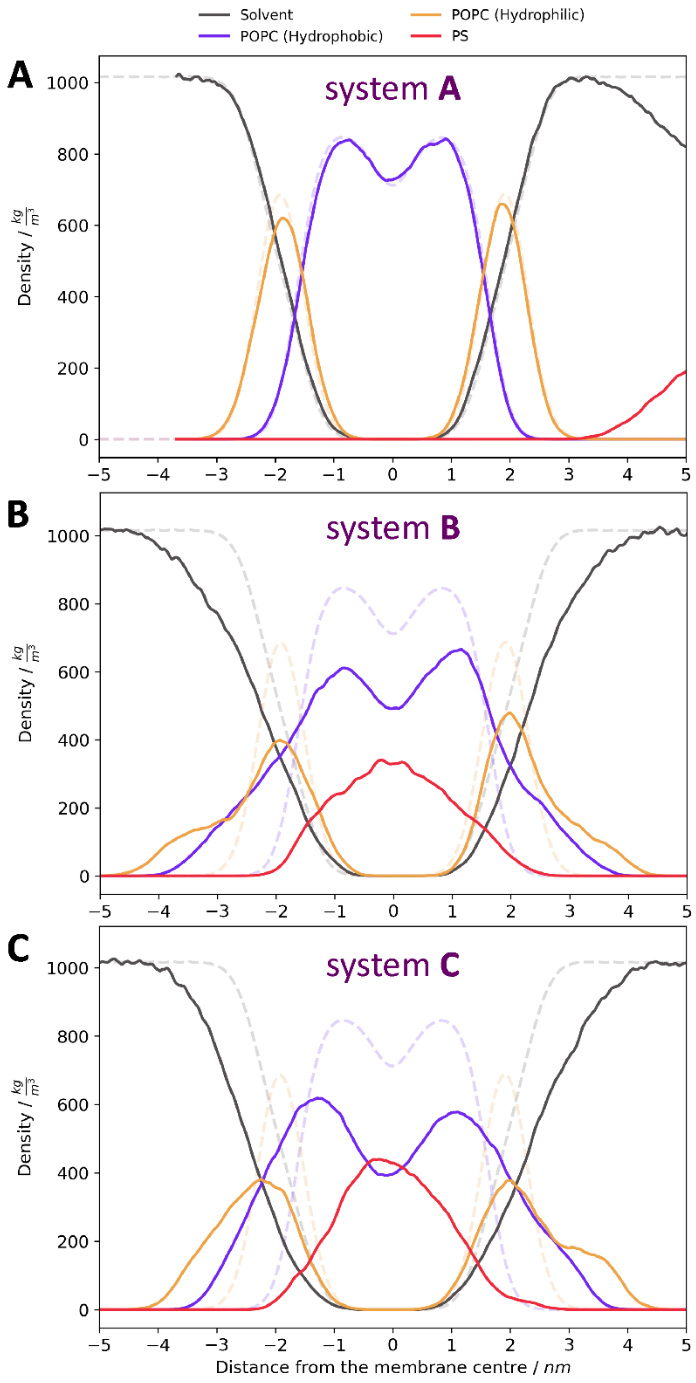

Figure S3. Mass density profiles of water, POPC headgroups and hydrocarbon chains, and PS coil in systems **A**, **B**, and **C**. Solid lines show the profiles at the end of the simulation. Transparent dashed lines depict values for the pure POPC membrane.

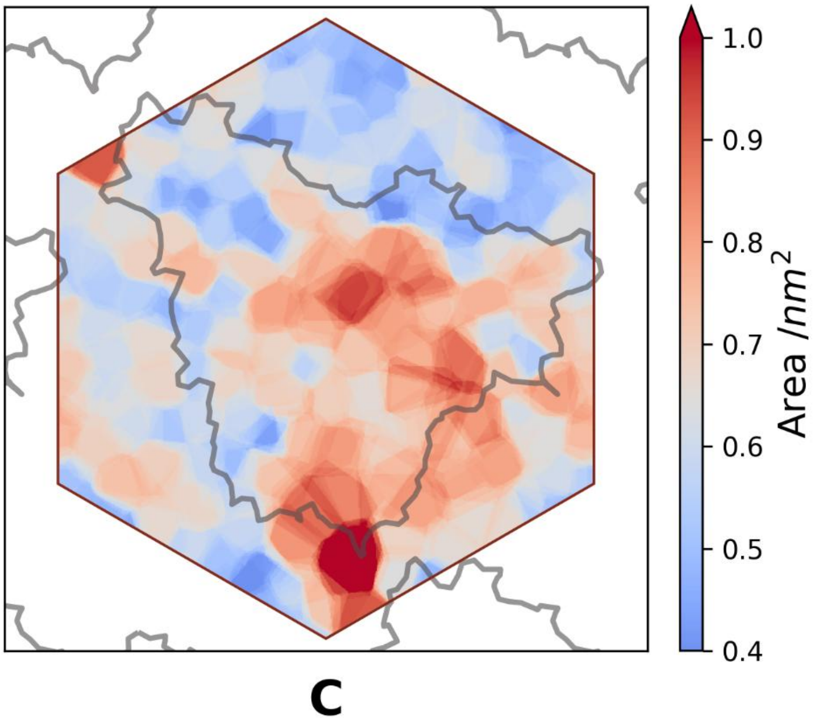

Figure S4. 2D maps of the area per lipid (APL) for system **C**. The maps were calculated by averaging the data over a 10 ns window at the end of the trajectory. The PS coil and its periodic images are projected onto the membrane surface and shown as a gray outline overlayed on the map. The color scale is centered at 0.64 nm^2, which corresponds to average APL of the POPC membrane. Unit cell boundaries are marked in burgundy.

ACKNOWLEDGMENT

This research was supported from the Anthropocene Priority Research Area budget within the framework of the "Excellence Initiative – Research University" program at Jagiellonian University. Simulations were carried out using computing resources of PLGrid, on the Prometheus, Ares, and Athena clusters. The work is supported under the Polish Ministry of Science and Higher Education project: “Support for research and development with the use of research infrastructure of the National Synchrotron Radiation Centre SOLARIS” under contract nr 1/SOL/2021/2.
